# Somatic cell score: gene polymorphisms and other effects in Holstein and Simmental cows

**DOI:** 10.5713/ab.20.0720

**Published:** 2021-04-23

**Authors:** Jindřich Čítek, Michaela Brzáková, Lenka Hanusová, Oto Hanuš, Libor Večerek, Eva Samková, Eva Jozová, Irena Hoštičková, Jan Trávníček, Martin Klojda, Lucie Hasoňová

**Affiliations:** 1 Faculty of Agriculture, South Bohemia University, CZ37005 Ceske Budejovice, Czech Republic; 2 Department Genetic and Animal Breeding, Institute of Animal Science, CZ104 00 Prague, Czech Republic; 3 Dairy Research Institute, CZ16000 Prague, Czech Republic

**Keywords:** Acyl-CoA Diacylglycerol Transferase 1 (*DGAT1*), Breed, Caseins, Dairy Cattle, Lactose, Milk

## Abstract

**Objective:**

The aim of the study was to evaluate the influence of gene polymorphisms and nongenetic factors on the somatic cell score (SCS) in the milk of Holstein (n = 148) and Simmental (n = 73) cows and their crosses (n = 6).

**Methods:**

The SCS was calculated by the formula SCS = log_2_(SCC/100,000)+3, where SCC is the somatic cell count. Polymorphisms in the casein alpha S1 (*CSN1S1*), beta-casein (*CSN2*), kappa-casein (*CSN3*), beta-lactoglobulin (*LGB*), acyl-CoA diacylglycerol transferase 1 (*DGAT1*), leptin (*LEP*), fatty acid synthase (*FASN*), stearoyl CoA desaturase 1 (*SCD1*), and 1-acylglycerol-3-phosphate O-acyltransferase 6 (*AGPAT6*) genes were genotyped, and association analysis to the SCS in the cow’s milk was performed. Further, the impact of breed, farm, year, month of the year, lactation stage and parity on the SCS were analysed. Phenotype correlations among SCS and milk constituents were computed by Pearson correlation coefficients.

**Results:**

Only *CSN2* genotypes A^1^/A^2^ were found to have significant association with the SCS (p<0.05), and alleles of *CSN1S1* and *DGAT1* genes (p<0.05). Other polymorphisms were not found to be significant. SCS had significant association with the combined effect of farm and year, lactation stage and month of the year. Lactation parity and breed had not significant association with SCS. The phenotypic correlation of SCS to lactose content was negative and significant, while the correlation to protein content was positive and significant. The correlations of SCS to fat, casein, nonfat solids, urea, citric acid, acetone and ketones contents were very low and not significant.

**Conclusion:**

Only *CSN2* genotypes, *CSN1S1* and *DGAT1* alleles did show an obvious association to the SCS. The results confirmed the importance of general quality management of farms on the microbial milk quality, and effects of lactation stage and month of the year. The lactose content in milk reflects the health status of the udder.

## INTRODUCTION

Mastitis is one of the most crucial health problems in the dairy industry. It causes immense financial damages by decreasing the milk yield, untimely culling of dairy cows, and increasing treatment costs. Clinical and subclinical mastitis reduces the milk quality, interferes with the processing of milk and has the potential to endanger human health due to antibiotic residues [1]. For instance, under the Czech Republic (CR) conditions, with every increase in the somatic cell count (SCC) by 10^5^ mL^−1^, milk production is reduced by 51 kg per cow per lactation on average. Fat and protein contents are decreased, as is the payment price [2–4]. In the CR, the economic loss was estimated at 410 US dollars (USD) per cow with mastitis, which is equal to revenue from sales for 950 liters of milk at the milk price of 0.43 USD per liter. In the total losses inflicted by mastitis participate the lower takings from the sale of milk by 53%, higher culling of cows (herd turnover, 20%), higher costs for drugs and treatments (14%), labor for the treatment of ill cows (7%) and penalties on the farmer’s milk price (6%).

There are various causes of mastitis. From the perspective of cow breeds, the heritability of mastitis occurrence is unfortunately very low. In the Czech dairy cattle population, heritabilities of 0.10 and repeatabilities of 0.19 at the most were found [5]. The health status of the mammary gland is often assessed indirectly by SCC or somatic cell score (SCS), as the genetic correlation between SCC and clinical mastitis is often significant [6]. However, the heritabilities of these indicators are also usually very low. In the Czech dairy population, the heritabilities of SCS were 0.10 to 0.11 in Simmental and slightly higher in Holstein, at 0.10 to 0.14, depending on the lactation stage [7]. A somewhat higher heritability of 0.19 was found in Brazilian Holstein [8].

Within this context, analyses of some major genes were performed, aimed at the identifying polymorphisms associated with udder health. The analyses are often performed together with those assessing milk performance [9]. The analysis focused inter alia on the polymorphisms in the mannose-binding lectin-associated serine protease 1 gene (*MASP1*). The authors found an association of g.5766A>G in the gene with milk protein percentage, but not with fat percentage, milk yield and SCS. Other authors found a significant impact of the polymorphisms in the lipocalin-2 (*LCN2*) gene on the average SCS of milk, but not on the milk yield, protein, fat and lactose contents, or the incidence of mastitis in cows [10,11]. These findings correspond with the fact that the LCN2 protein is secreted inter-alia by neutrophils. Another source reported the results of the analysis of three polymorphisms in the fatty acid desaturase 2 (*FADS2*) gene [12]. The enzyme plays a pivotal role in the biosynthesis of polyunsaturated fatty acids, and previous studies provided evidence that *FADS2* was one of the most downregulated genes during negative energy balance in the liver of postpartum dairy cattle. The polymorphisms in the gene were significantly associated with test-day milk yield, fat percentage and 305-day milk, fat and protein yields, protein percentage and SCS in the investigated population. Also the associations between polymorphisms in the gene and fatty acids contents were found [13,14].

Obviously, there are some major genes with the potential to change milk production and to improve udder health and resistance against mastitis. Other authors have performed whole-genome searches and have identified a few regions, SNPs and genes associated with the indicators of infectious diseases incl. mastitis [15–17]. Next-generation sequencing enabled the establishment of a candidate gene set of 48 genes associated with mastitis in Holstein cattle [18].

The aim of this study was to evaluate the impact of different factors on the SCS in milk of Holstein and Simmental cows. The polymorphisms in the casein alpha S1 (*CSN1S1*), beta-casein (*CSN2*), kappa-casein (*CSN3*), beta-lactoglobulin (*LGB*), acyl-CoA diacylglycerol transferase 1 (*DGAT1*), leptin (*LEP*), fatty acid synthase (*FASN*), stearoyl CoA desaturase 1 gene (*SCD1*) and 1-acylglycerol-3-phosphate O-acyltransferase 6 (*AGPAT6*) genes were genotyped, and an association analysis was performed.

## MATERIALS AND METHODS

### Animals

All animal experiments were under supervision of the Institutional Animal Care and Use Committee of the Faculty of Agriculture of South Bohemia University, where the experiment was carried out, with approval number 22036/2019-MZE-18134. DNA was extracted noninvasively from milk samples.

The group analyzed (n = 227) consisted of cows of Holstein (n = 148) and Simmental (n = 73) breeds in the Czech Republic, and their crosses (n = 6). The cows were stabled in five farms in free stall housing (n_1_ = 49; n_2_ = 31; n_3_ = 56; n_4_ = 50; n_5_ = 41). The cows calved in 2015 through 2017, and the milk samples were obtained repeatedly within two following lactations. The cows were in the 1st up to 6th lactation and were sampled throughout the year. The number of samples from one cow varied from one to five. The feed ratio consisted of maize silage, grass silage, hay and feed concentrates year-round.

### Sampling and milk analyses

The individual cow milk samples were treated (preserved) with DF Control Microtabs tableted preservative preparate and 0.03% bronopol, transported under cold conditions (<8°C) to the laboratory and analyzed for SCC. The analysis was performed in an accredited [19] laboratory for milk analysis that is owned by the Czech Moravian Breeder’s Corporation on SCC (10^3^ mL^−1^) using a Somacount flow cytometer (Bentley Instruments, Chaska, MN, USA). The analysis was based on the photometry measurement of the complex reaction from the reaction between SCC DNA and ethidium bromide. These instruments were regularly calibrated according to relevant SCC reference values by using the so-called direct microscopic method and were also included in proficiency testing with regularly successful results [19–21]. The extended result uncertainty (95% probability level) was ±9.3% for SCC ≤900 10^3^ mL^−1^.

The milk composition, i.e., the contents of fat, crude protein, casein, lactose monohydrate, nonfat solids (NFS), urea, citric acid, acetone, and ketone as beta hydroxybutyrate, was determined in laboratories of the Czech Moravian Breeder’ s Corporation. Infrared spectroscopy by filter technology and by Fourier data transformation was applied. The instrumentation of Foss Electric (Hilleroed, Denmark) and Bentley Instruments (Chaska, MN, USA) was used. The instruments went through proficiency testing with regular successful results. The extended result uncertainties (95% probability level) were ±2.77% for fat (0.101% for original unit as a gram per 100 grams), ±2.59% for crude (total nitrogen content×6.38) protein (0.085%), and ±2.77% for lactose monohydrate (0.115%).

### Genotyping

DNA was isolated from the milk samples using a MagCore HF16 Plus DNA/RNA extractor (RBC Bioscience, New Taipei, Taiwan). Genotyping was performed by the PCR/RFLP method. *CSN1S1* gene alleles B and C were genotyped according to the methods of Ardicli et al [22] and Kučerová et al [23]; *CSN2* gene alleles A and B as in Medrano and Sharrow [24]; alleles A^1^ and A^2^ according to Miluchová et al [25]; *CSN3* gene alleles A, B, C and E according to the methodology of Barroso et al [26]; *LGB* gene alleles A and B according to the methods of Strzalkowska et al [27]; *DGAT1* gene alleles A (alanine) and K (lysine) as in Kuhn et al [28]; *LEP* gene alleles M and W as in Buchanan et al [29]; *FASN* gene alleles A and G according to Roy et al [30]; and *SCD1* gene alleles C and T according to the methods of Inostroza et al [31]. *AGPAT6* gene alleles C and T were genotyped by using fragment analysis as in Littlejohn et al [22–32]. The sequences of primers used in the PCR and restriction endonucleases used for genotyping are given in Supplementary Table S1. The genotype and allelic frequencies were calculated (Table 1).

### Statistical analysis

Somatic cell score was calculated by the formula:


$$\text{SCS}={\text{log}}_{2}\;(\text{SCC}/100,000)+3$$

where SCC, somatic cell count.

This method of evaluating individual SCCs is based on the work by Ali and Shook [33] and Shook [34]. The advantage of this evaluation lies in the normalization of the frequency distribution of SCC data for various statistical evaluations using parametric methods. The topic was further elaborated until the emergence of this transformation equation for recalculation of individual dairy cow SCCs on a linear score based on SCCs (SCSs) on a log-2 basis [35,36]. The main advantage of the SCS is the linearization of the SCC relationship to milk yield losses of dairy cows, mainly due to the occurrence of subclinical mastitis. The SCS scale was then used in scientific work as well as in practical breeding programs as a very suitable characteristic to control the dynamics of SCC development [37–42].

Statistical analyses were performed using SAS (SAS 9.3, SAS Institute, Cary, NC, USA). The data set contained repeated measurements of SCC transformed to SCS per cow obtained in the two following lactations. To analyze the effects of polymorphisms and other effects on the SCS, the linear mixed model (MIXED procedure of the SAS system with repeated measurements) and the least squared mean method were used. The model for the evaluation of multiple effect of all gene polymorphisms, breed and non-genetic effects was developed as follows:


$$\begin{array}{ll} {\text{SCS}}_{\text{ijklmn}}=\; & \mu +CSC1S1_{\text{i}}+CSN2{AB}_{j}+CSN2A1A2_{k}+CSN3_{\text{l}}\\ & +{LGB}_{m}+{DGAT1}_{\text{n}}+{LEP}_{\text{o}}+{FASN}_{\text{p}}+SCD1_{\text{q}}\\ & +AGPAT6_{\text{r}}+{HY}_{\text{s}}+{\text{lac}}_{\text{t}}+{\text{month}}_{\text{u}}+{\text{parity}}_{\text{v}}+{\text{breed}}_{\text{w}}\\ & +{\text{sire}}_{\text{x}}+{\text{cow}}_{\text{y}}+{\text{e}}_{\text{ijklmnopqrstuvwxy}} \end{array}$$

where SCS_ijklmn_, somatic cell score; *CSC1S1*_i_, fixed effect of genotype *CSC1S1*_i_ (class effect i = 1, 2); *CSN2AB*_j_, fixed effect of genotype *CSN2AB* (class effect j = 1, 2, 3); *CSN2A1A2*_k_, fixed effect of genotype *CSN2A1A2* (class effect k = 1, 2, 3); *CSN3*_l_, fixed effect of genotype *CSN3* (class effect l = 1,…, 5); *LGB*_m_, fixed effect of genotype *LGB* (class effect m = 1, 2, 3); *DGAT1*_n_, fixed effect of genotype *DGAT1* (class effect n = 1, 2); *LEP*_o_, fixed effect of genotype *LEP* (class effect o = 1, 2, 3); *FASN*_p_, fixed effect of genotype *FASN* (class effect p = 1, 2); *SCD1*_q_, fixed effect of genotype *SCD1* (class effect q = 1, 2, 3); *AGPAT6*_r_, fixed effect of genotype of *AGPAT6* (class effect r = 1, 2, 3); HY_s_, combined fixed effect of farm and year (class effect s = 1, …, 5); lac_t_, fixed effect of the lactation stage (class effect t = 1, 2, 3 for day in milk 1 to 99, 100 to 200, 201 to 305); month_u_, fixed effect of the calendar month of the year according to the sampling (class effect u = 1, …, 10); parity_v_, fixed effect of lactation parity (class effect v = 1,…, 6); breed_w_, fixed effect of breed (class effect w = 1, 2, 3,); sire_x_, random effect of the father of the cow; cow_y_, permanent environment of the cow (repeated measurement); and e_ijklmnopqrstuvwxy_, random residual effect.

Only cows with all genotypes were involved into the computation. The model for the effect of allele was the same as for the genotypes, just there was allele instead of corresponding gene, when allele was fixed effect (class effect l = 1, 2, 3 for *CSN3* alleles, 1, 2 for other alleles).

For post hoc comparisons, the Tukey-Kramer test was used [43].

Phenotype correlations among traits were computed by Pearson correlation coefficients (the CORR Procedure, SAS 9.4). The correlations among SCS on the day of sampling and the percentages of lactose, fat, protein, casein, NFS and urea on the day of sampling were computed involving all measurements.

## RESULTS AND DISCUSSION

Some of the included genes have been studied for a long time, such as caseins, while others have only been studied for a brief time. The analyses were mostly focused on the relation to milk or meat performance, including quality. In this work, we analyzed the impact of the polymorphisms on the microbial quality of the milk; other factors were also tested.

As shown in Table 2, a significant effect of gene polymorphisms on the SCS was rare. Other than genotypes in *CSN2* at p<0.05, the other genes did not show an impact on the SCS. Similarly, the effect of alleles was mostly nonsignificant. Only the differences between B and C alleles in the *CSN1S1* gene, A and K alleles in the *DGAT1* gene were significant at p<0.05. In *CSN1S1* gene, B allele was better (lower SCS), and the genotype BB was better than BC as well, even though nonsignificantly. Regarding *DGAT1* gene polymorphisms, the genotype and allele differences had the same tendency, i.e., genotype AA was nonsignificantly better than genotype KA, and allele A was better than allele K. In the *CSN2* gene, genotype A^1^A^1^ was worse than genotypes A^1^A^2^ and A^2^A^2^, but the tendency of the allele effect was opposite. However, the difference between alleles was nonsignificant and thus must be interpreted cautiously. For *DGAT1*, Sanders et al [44] reported the influence of K and A alleles and polymorphism in the promoter on the SCS, but their haplotypes did not show a significant impact. In addition, the interaction of the polymorphisms did not affect the SCS. The authors did not find a dominance effect of KA polymorphism on the SCS. Other authors did not find significant differences among *DGAT1* genotypes, but the effect of allele substitution was significant [45]. Others refer to the significance of a *DGAT1* allele substitution effect for the milk and fat yields and fat and protein percentages, but not for the SCS [46]. Another source refers to the significant influence of the *CSN3* polymorphisms on the SCS [47]. However, in this context, the relationship of the SCS and the occurrence of clinical mastitis must be mentioned. In some cases, even when the impact of the polymorphisms on some genes on the SCS was stated, their effect on mastitis occurrence was not found [10,48].

To eliminate false positive results, also the Bonferroni correction was used, when the significancy threshold is divided by the number of genes in the analysis, the p value must be <0.005 in our case. This criterion was not met in any polymorphism analysed, it confirms the insignificancy of their effect on the SCS in cow’s milk. As the number of analyses from each cow varied from one to five, we made the analysis excluding the cows with only one sample. The results were only negligibly different from those from the all dataset (data not shown).

**Figure 1:**
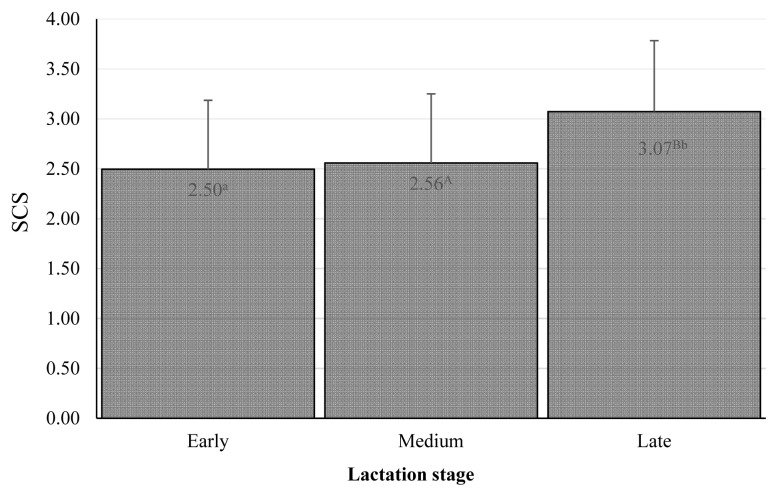
Somatic cell score among lactation stages. The values in columns are means, the bars are standard deviations, SCS is somatic cell score. Early is day 1 to 100, medium day 101 to 200, late day 201 to 305. ^A,B^ The differences are significant at p<0.01. ^a,b^ The differences are significant at p<0.05.

**Figure 2:**
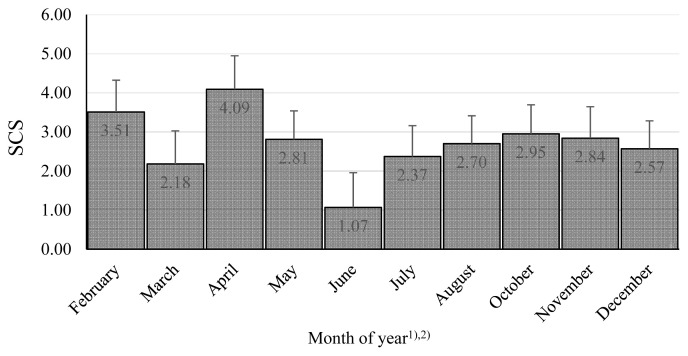
Somatic cell score among months during years. The values in columns are means, the bars are standard deviations, SCS is somatic cell score. ^1)^ There were no milk samples in January and September. ^2)^ The differences February vs March; April vs March, May, July, August, December; June vs November, December are significant at p<0.05. The differences June vs February, April, May, August, October are significant at p<0.01.

The combined effect of farm and year was significant, and the differences were substantial (Table 3). These findings emphasize the importance of conditions in particular dairy farms, although in our research, the system of farming was similar: the cows were kept in free stall barnss and were fed by conserved fodder in all farms. At the beginning of lactation, the SCS was lowest, and thereafter, it showed increases in the 2nd and 3rd phases, where the effect of lactation stage was significant (Figure 1). Similarly, the month of the year was significant, as there was a clear tendency for worse microbial quality in April, with the best in June to August (Figure 2). Many authors stated, that season as well as month had a significant effect on the SCC values in bulk milk [49–51]. Their results demonstrated that mastitis risks rise with increasing age or parity and during summer, late spring and early autumn [49]. Similarly Arsoy [51] found higher values of SCC in the summer months, although there are also works where they found the opposite results [50].

**Figure 3:**
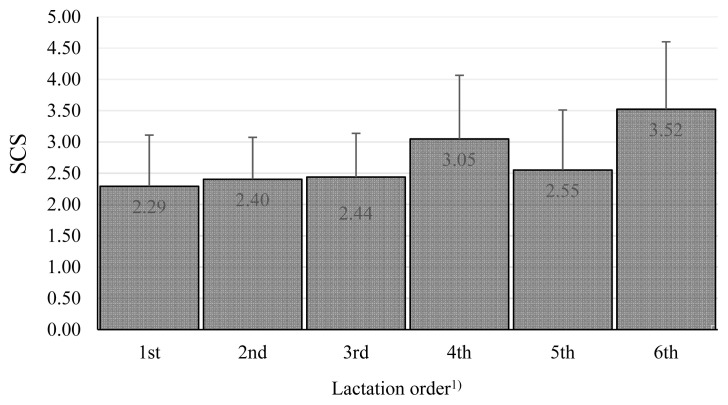
Somatic cell score among lactation orders. The values in columns are means, the bars are standard deviations, SCS is somatic cell score. ^1)^ The effect of lactation order was nonsignificant.

We also tested other effects. Breed and lactation parity (Figure 3) were not found to be significant.

Finally, we evaluated the phenotypic correlations of SCS to milk constituents (Table 4). The correlation with lactose content was negative and significant, while the correlation to protein was positive and significant. The correlations of SCS to casein, NFS and urea contents were positive, low and nonsignificant, and the correlations to ketones were negative, low and nonsignificant. The correlations of SCS to citric acid and acetone were also negative, low and nonsignificant, but the p values were nearing the significance threshold. This was similar to the correlation of SCS to fat percentage, but its correlation was positive. The significant relationship between the SCS and some constituents may indicate that the udder responds to the changing health status. By all means, it is applicable for the lactose percentage. Its correlation coefficient is relatively high and significant. As confirmed by other authors, the worsening health status of the mammary gland reflects the change in lactose content [52]. The authors point to the decreasing dry matter content by reducing the milk lactose and the fat, casein, and calcium in the milk of cows with subclinical mastitis. Conversely, the content of whey protein increases, as does the SCC. Mastitis is genetically correlated with lactose yield, and as the amount of the synthesized lactose is the key regulator of milk volume, this result confirms that high-producing cows are more genetically susceptible to mastitis [53,54]. Therefore, the lactose content in milk could be potentially used as an indicator to improve udder health.

## CONCLUSION

Generally, the impact of ten polymorphisms in nine genes was weak; only *CSN2* genotypes A^1^/A^2^ showed significance, and alleles of *CSN1S1* and *DGAT1*. The importance of farm management for milk quality was confirmed, as was the importance of lactation stage and month of the year. The lactose content in milk is a good indicator of changed health status of the mammary gland.

## Figures and Tables

**Table 1:** Frequencies of genotypes and alleles

Gene	Genotype	n^1)^	Relative frequencies	χ^2^	Allele	Allele frequencies
*CSN1S1*	BB	201	88.55	0.367^ns^	B	0.943
	BC	26	11.45		C	0.057
*CSN2*	AA	9	3.96	0.269^ns^	A	0.220
	AB	82	36.12		B	0.780
	BB	136	59.91			
*CSN2*	A^1^A^1^	23	10.85	1.956^ns^	A^1^	0.283
	A^1^A^2^	74	34.91		A^2^	0.717
	A^2^A^2^	115	54.25			
*CSN3*	AA	100	44.05	3.408^ns^	A	0.676
	AB	98	43.17		B	0.284
	AE	9	3.96		C	0.004
	BB	12	5.29		E	0.035
	BC	2	0.88			
	BE	5	2.20			
	EE	1	0.44			
*LGB*	AA	8	3.52	39.725^**^	A	0.436
	AB	182	80.18		B	0.564
	BB	37	16.30			
*DGAT1*	AA	211	92.95	0.133^ns^	A	0.965
	KA	16	7.05		K	0.035
*LEP*	MM	145	75.52	1.067^ns^	M	0.862
	MW	41	21.35		W	0.138
	WW	6	3.13			
*FASN*	AG	60	26.55	2.338^ns^	A	0.133
	GG	166	73.45		G	0.867
*SCD*	CC	67	29.52	4.857^ns^	C	0.590
	TC	134	59.03		T	0.410
	TT	26	11.45			
*AGPAT6*	CC	69	31.36	21.560^**^	C	0.648
	TC	147	66.82		T	0.352
	TT	4	1.82			

*CSN1S1*, casein alpha S1; *CSN2*, beta-casein; *CSN3*, kappa-casein; *LGB*, beta-lactoglobulin; *DGAT1*, acyl-CoA diacylglycerol transferase 1; *LEP*, leptin; *FASN*, fatty acid synthase; *SCD*, stearoyl CoA desaturase; *AGPAT6*, 1-acylglycerol-3-phosphate O-acyltransferase 6;

ns , Nonsignificant.

1) Number of animals with respective genotypes.

** Significant differences between genotype frequencies calculated on the basis of Hardy-Weinberg equilibrium and empirical frequencies (p<0.01).

**Table 2:** Somatic cell score according to genotype

Gene	Genotype	n^1)^	LSM±SE	p-value	

				Effect of genotype	Effect of allele
*CSN1S1*	BB	261	2.519±0.653	0.303	0.030^*^
	BC	39	2.898±0.763		B<C
*CSN2*	AA	7	1.994±1.041	0.397	0.981
	AB	108	3.092±0.630		
	BB	185	3.040±0.619		
*CSN2*	A^1^A^1^	27	3.407^A^^a^±0.771	0.019^*^	0.263
	A^1^A^2^	110	2.206^B^±0.696		A^1^<A2^2^
	A^2^A^2^	163	2.512^b^±0.685		
*CSN3* ^2)^	AA	124	2.800±0.691	0.725	A:B 0.754
	AB	141	2.511±0.674		A:E 0.103
	AE	11	3.078±0.858		B:E
	BB	14	2.408±0.841		
	BE	10	2.746±0.841		
*LGB*	AA	15	2.658±0.815	0.819	0.921
	AB	234	2.833±0.712		
	BB	51	2.635±0.711		
*DGAT1*	AA	274	2.644±0.692	0.770	0.024^*^
	KA	26	2.773±0.748		A<K
*LEP*	MM	217	2.856±0.685	0.786	0.975
	MW	73	2.696±0.682		
	WW	10	2.574±0.885		
*FASN*	AG	89	2.609±0.728	0.454	0.618
	GG	211	2.809±0.668		
*SCD1*	CC	94	2.796±0.694	0.877	0.937
	TC	172	2.732±0.676		
	TT	34	2.598±0.766		
*AGPAT6*	CC	96	2.984±0.631	0.387	0.883
	CT	200	3.168±0.583		
	TT	4	1.975±1.154		

LSM, least squared mean; SE, standard error; *CSN1S1*, casein alpha S1; *CSN2*, beta-casein; *CSN3*, kappa-casein; *LGB*, beta-lactoglobulin; *DGAT1*, acyl-CoA diacylglycerol transferase 1; *LEP*, leptin; *FASN*, fatty acid synthase; *SCD1*, stearoyl CoA desaturase 1; *AGPAT6*, 1-acylglycerol-3-phosphate O-acyltransferase 6.

1) n number of samples from cows with a particular genotype.

2) for *CSN3* BC and EE genotypes the number of samples was too low to compute LSM, the same for comparison of the effect of B and E alleles.

* Significant at p<0.05;

** significant at p<0.01.

a,b Different letters between genotypes in the same column represent significant differences at p<0.05.

A,B Different letters between genotypes in the same column represent significant differences at p<0.01.

**Table 3:** Effect of different factors on the somatic cell score (p-values)

Factor	p-value
Farm×year (HY)	0.020^*^
Lactation stage^1)^	0.022^*^
Lactation parity	0.803
Month of the year	0.027^*^
Breed^2)^	0.752

1) The lowest in 1st part of the lactation, the highest in the 3rd part.

2) Milk of Simmental cows was better than that of Holstein and crossbred cows.

* Significant at p<0.05.

**Table 4:** Pearson correlations for somatic cell score and content of milk constituents on the day of sampling

Items	n^1)^	Correlation	p-value
Lactose (%)	434	−0.280	<0.0001^**^
Fat (%)	433	0.0825	0.087
Protein (%)	434	0.156	0.001^**^
Casein (%)	184	0.074	0.319
Nonfat solid (%)	331	0.053	0.337
Urea (mg/100 mL)	434	0.001	0.983
Citric acid (%)	433	−0.088	0.066
Acetone (%)	433	−0.092	0.056
Ketones (mmol/L)	430	−0.070	0.150

1) n number of samples from cows with measurements for the trait.

** Significant at p<0.01.

## SUPPLEMENTARY MATERIAL

Sequences of primers used in the polymerase chain reactions and restriction endonucleases used for genotyping
