# Supplementary material for: Somatic cell score: gene polymorphisms and other effects in Holstein and Simmental cows
Source: Anim Biosci. 2021 Apr 23;35(1):13–21. doi: 10.5713/ab.20.0720 (PMC8738924; doi:10.5713/ab.20.0720)
Supplement: Supplementary file 1 [file ab-20-0720-suppl.pdf]

Table S1. Sequences of primers used in the polymerase chain reactions and restriction endonucleases used for genotyping

Locus	Primer	Sequence	Restriction endonuclease
CSN1S1	Forward	5’ACAATTCTACCAGCTGGATGCCTATC3’	*Hph*I
	Reverse	5’CACGCTCCACAGTTCCTGAGTAA3’	
CSN2 (A, B)	Forward	5´- CCA GAC ACA GTC TCT AGT CTA TCC C – 3´	*Hsp*I
	Reverse	5´- CAA CAT CAG TGA GAG TCA GGC TCC G – 3´	
*CSN2 (*A^{1}, A^{2}*)*	Forward	5´- CCT TCT TTC CAG GAT GAA CTC CAG G – 3´	*Dde*I
	Reverse	5´- GAG TAA GAG GAG GGA TGT TTT GTG GGA GGC TCT – 3´	
CSN3	Forward	5´- TGT GCT GAG TAG GTA TCC TAG TTA TGG – 3´	*Hinf*I, *Hae*III,
	Reverse	5´- GCG TTG TCT TCT TTG ATG TCT CCT TAG – 3´	*Mae*II
LGB	Forward	5´- TGT GCT GGA CAC CGA CTA CAA AAA G – 3´	*Hae*III
	Reverse	5´- GCT CCC GGT ATA TGA CCA CCC TCT -3´	
DGAT1	Forward	5´ - GCA CCA TCC TCT TCC TCA AG – 3´	*Cfr*I
	Reverse	5´- GGA AGC GCT TTC GGA TG – 3´	
LEPTIN	Forward	5´- ATG CGC TGT GGA CCC CTG TAT C – 3´	*Hph*I
	Reverse	5´- TGG TGT CAT CCT GGA CCT TCC – 3´	
FASN	Forward	5´- AGA GCT GAC GGA CTC CAC AC – 3´	*Msc*I
	Reverse	5´- GCC GAT GCA CTC GAT GTA G – 3´	
SCD1	Forward	5´- ACC TGG CTG GTG AAT AGT GCT – 3´	*Fnu4H*I
	Reverse	5´- TCT GGC ACG TAA CCT AAT ACC CT- 3´	
AGPAT6	Forward	5´- CAA GGC GGC GTA GAC AAA – 3´	Fragment
	Reverse	5´- AGC CCC GTC AGA GGT TCA T – 3´	analysis
